# A Rare Case of Salmonella Enteritidis Sinusitis

**DOI:** 10.7759/cureus.75504

**Published:** 2024-12-10

**Authors:** Kelvin Yong Jie Lim, Ying Ying Chen, Wei Zhong Jonathan Chia, Ming Yann Lim, Stephanie Sutjipto

**Affiliations:** 1 Otorhinolaryngology, Tan Tock Seng Hospital, Singapore, SGP; 2 Infectious Disease, National Centre for Infectious Disease, Singapore, SGP; 3 Pathology, Tan Tock Seng Hospital, Singapore, SGP; 4 Infectious Disease, Tan Tock Seng Hospital, Singapore, SGP

**Keywords:** head and neck cancer pathology, infectious disease pathology, salmonella infection, sinonasal malignancy, squamous cell carcinoma (scc)

## Abstract

Nontyphoidal *Salmonella* is a common cause of gastroenteritis but can also lead to bacteremia and extraintestinal infections, including meningitis (more frequent in children and infants), endovascular infections (e.g., endocarditis and infected aneurysms), urinary tract infections, and bone or bone marrow infections (e.g., septic arthritis and osteomyelitis). However, ENT complications are rare.

We present the first-ever case of *Salmonella *Enteritidis sinusitis. A 77-year-old woman experienced worsening right facial swelling and pain persisting for one month. Upon examination, she exhibited right cheek swelling with induration, warmth, and redness extending to the infraorbital region. Computed tomography (CT) scan findings revealed a heterogeneous mass in the right maxillary sinus with evidence of locoregional destruction. Additionally, an abscess was detected in the right buccal space. During surgery, the right maxillary sinus was found to contain pink frond-like tissue and white-grey concretions. Histological examination revealed squamous cell carcinoma (SCC). Magnetic resonance imaging (MRI) showed enlarged right cervical lymph nodes, raising suspicion for metastatic nodal spread.

Further investigation indicated the presence of *Salmonella enterica *serovar Enteritidis in tissue cultures. The patient was ultimately diagnosed with stage IVA cT3N2bM0 right maxillary sinus squamous cell carcinoma (SCC) with concomitant *Salmonella* Enteritidis sinusitis.

Prior to the first surgery, she received treatment with amoxicillin-clavulanate for eight days, followed by six weeks of ciprofloxacin (culture-directed treatment) and two weeks of metronidazole to cover for anaerobes. Subsequently, she underwent a total maxillectomy, neck dissection and reconstruction utilizing a free anterolateral thigh flap, and adjuvant radiotherapy and is recovering well.

We discuss the possible mechanism of *Salmonella* Enteritidis infection in relation to kombucha intake.

## Introduction

We present a case report of an elderly woman who presented with right facial swelling and pain one month after a dental procedure. Examination revealed cheek swelling with induration, warmth, and redness extending to the infraorbital region, while computed tomography (CT) showed a destructive mass in the right maxillary sinus with abscess formation, orbital floor erosion, and extension into adjacent spaces. Nasoendoscopy revealed mucopus, and a biopsy of frond-like sinus tissue confirmed squamous dysplasia with invasive squamous cell carcinoma (SCC). Cultures from intraoperative specimens grew *Salmonella enterica *serovar Enteritidis, identified via matrix-assisted laser desorption/ionization-time of flight (MALDI-TOF).

Only three human cases of *Salmonella *sinusitis have been reported in the literature [1-3]. This manuscript provides a detailed description of a unique and rare case of *Salmonella *Enteritidis sinusitis, which, to the best of our knowledge, is the first documented instance of this condition. Additionally, we explore the potential causal relationship between her sinusitis and her consumption of kombucha, offering insights into the possible transmission route. This case highlights the importance of considering atypical pathogens such as *Salmonella *in sinusitis, particularly in immunocompromised individuals, and underscores the need for further research into unusual transmission routes.

## Case presentation

**Figure 1:**
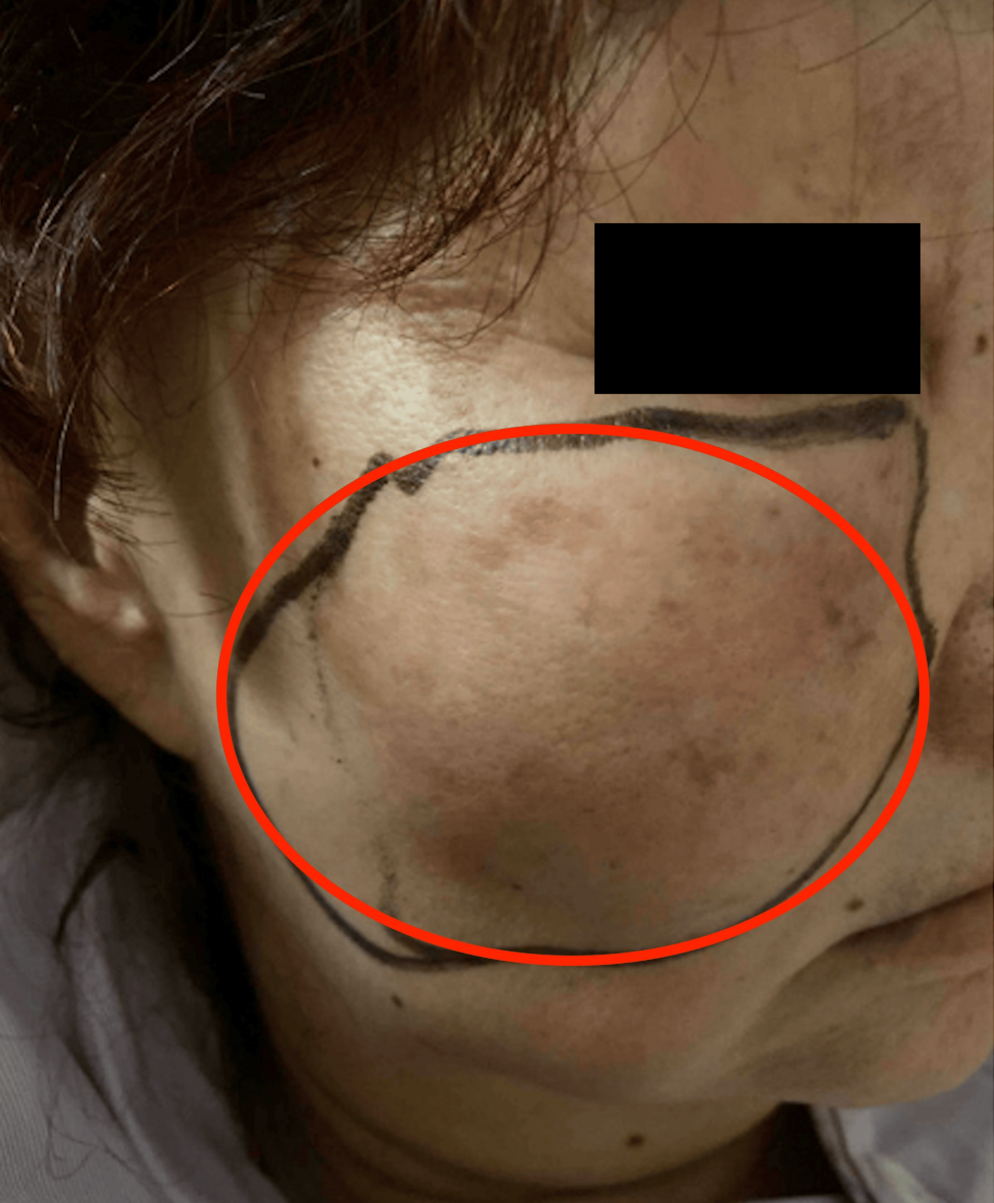
Clinical photo showing right cheek swelling

**Figure 2:**
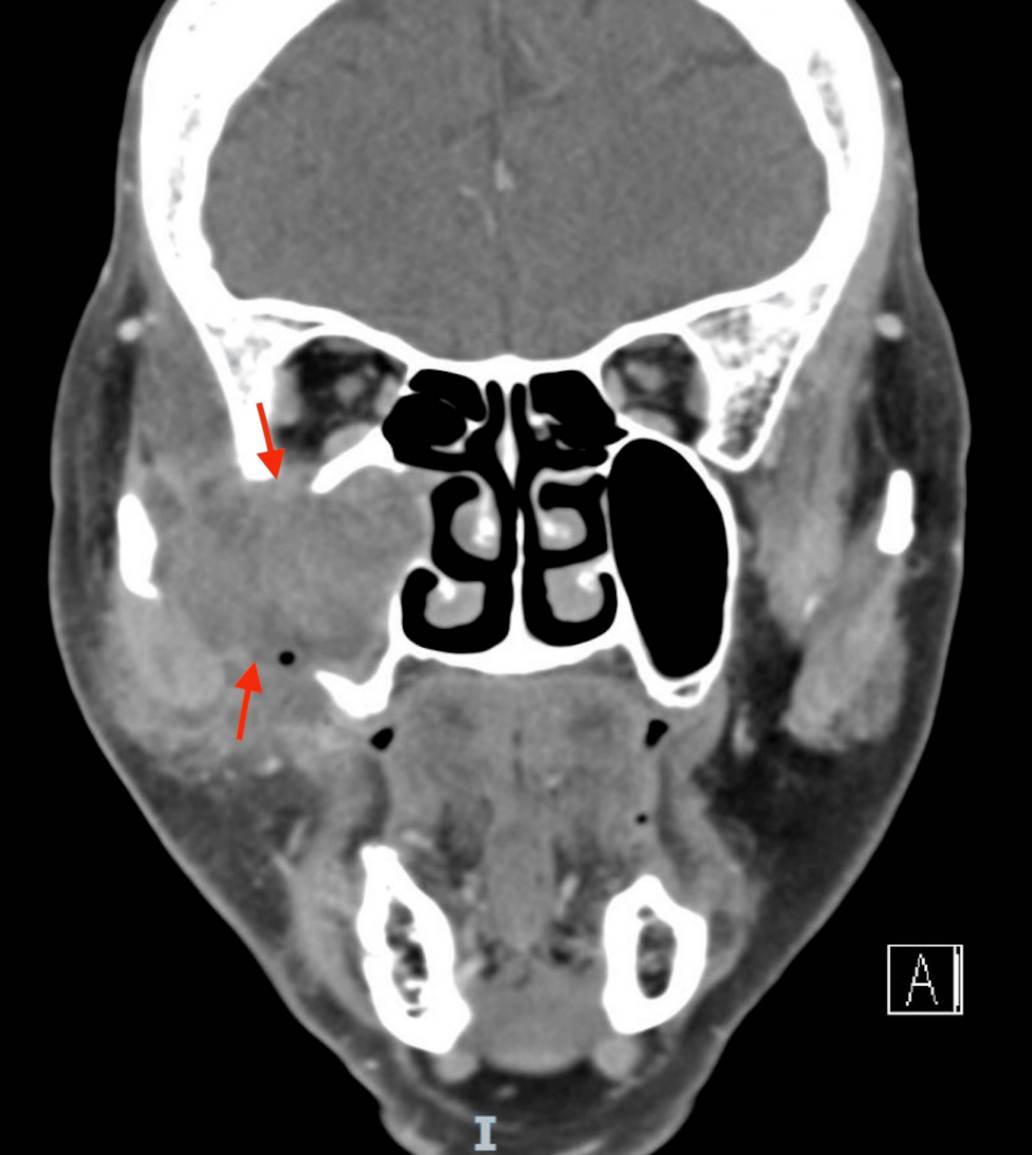
CT of the face depicting a heterogeneously enhancing soft tissue opacification with areas of rim enhancement, involving the right maxillary sinus, associated with the destruction of the anterior and lateral maxillary sinus with extension into the pre-antral fat, buccal and masticator spaces, and the floor of orbit erosion CT: computed tomography

A 77-year-old woman with a history of dyslipidemia presented with worsening right facial swelling and pain one month after a dental procedure. Examination revealed right cheek swelling with induration, warmth, and redness extending to the infraorbital region, as seen in Figure 1. No intraoral swelling or discharge was observed, and extraocular movements, vision, and facial sensation were intact. A CT scan of the face showed a heterogeneous mass in the right maxillary sinus with the destruction of the right lateral maxillary wall and extension into the right orbital floor, pre-antral fat, and buccal and masticator spaces with some areas of rim enhancement, as seen in Figure 2.

**Figure 3:**
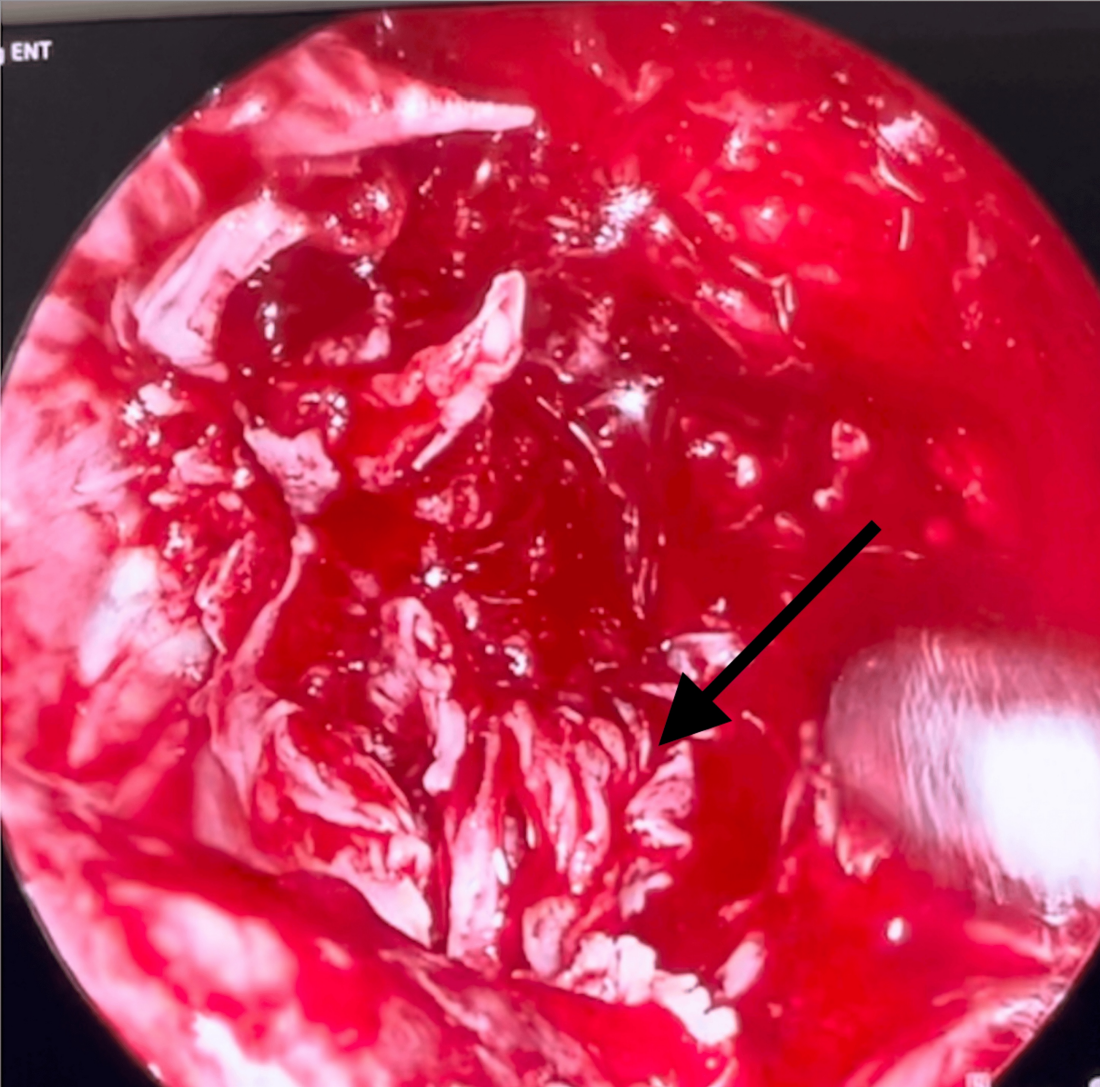
Endoscopic view of the right maxillary sinus depicting pink tissue with frond-like texture, interspersed with white-grey concretions

**Figure 4:**
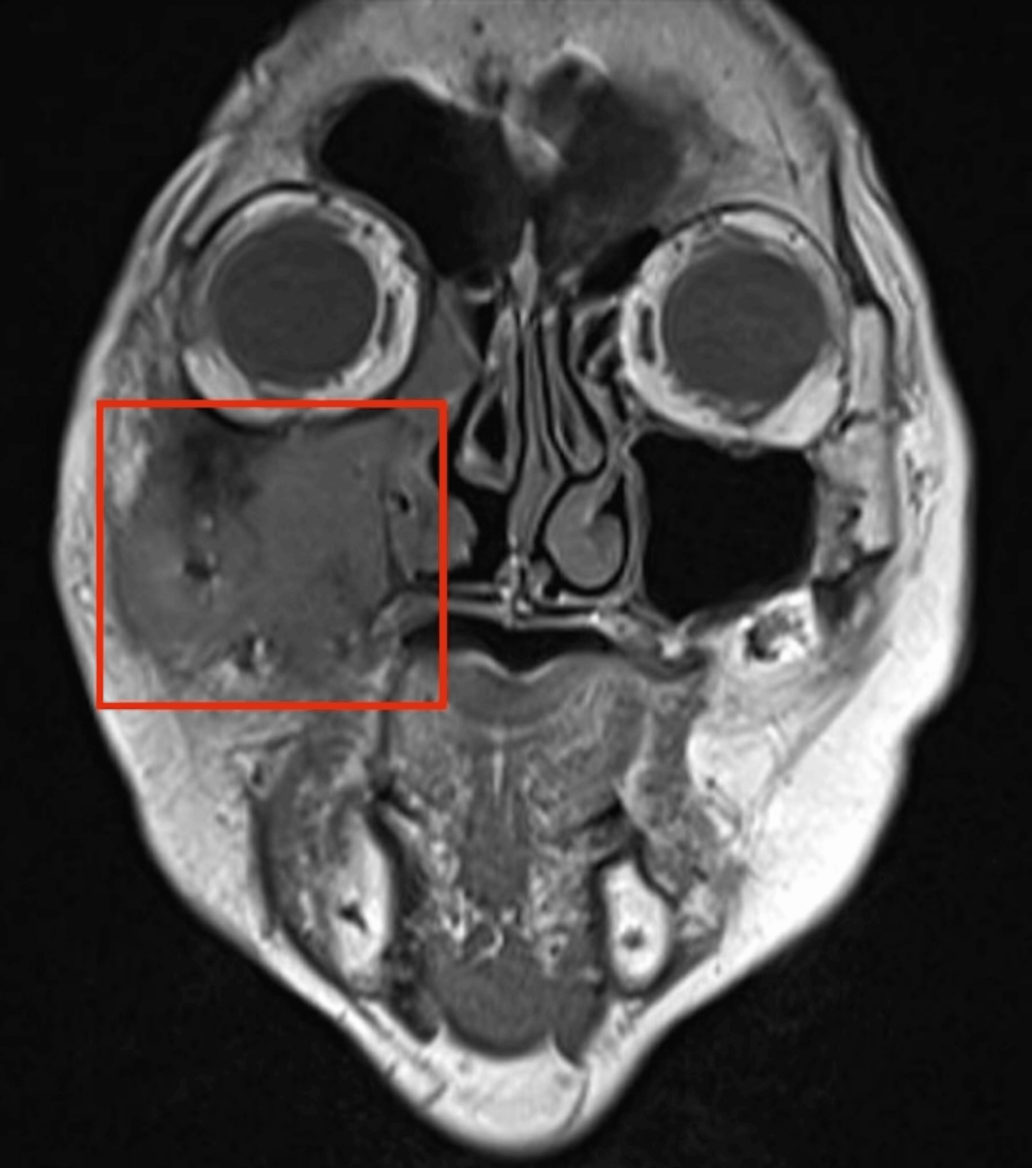
T1-weighted coronal MRI depicting a lobulated hypointense mass in the right maxillary sinus causing the destruction of the anterior and lateral maxillary walls, with extension into the pre-antral fat and buccal and masticator spaces MRI: magnetic resonance imaging

**Figure 5:**
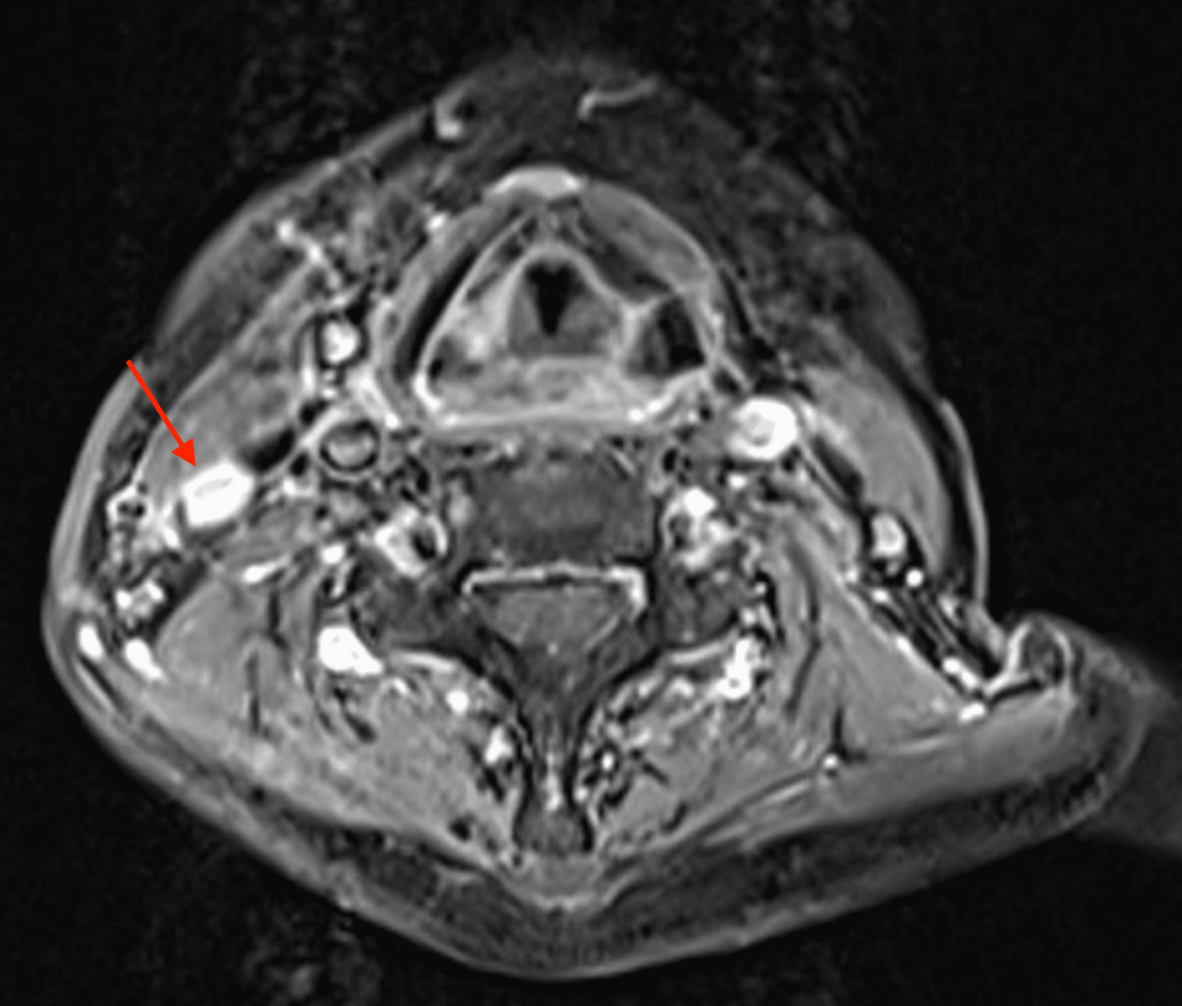
MRI of the neck depicting lymph node metastases to the right at level III, corresponding to an increased FDG uptake on PET-CT as in Figure 6 MRI, magnetic resonance imaging; FDG, fluorodeoxyglucose; CT, computed tomography

**Figure 6:**
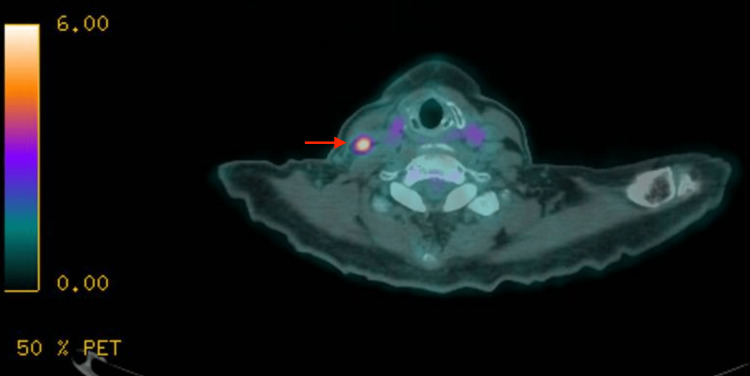
PET-CT depicting lymph node metastasis to the right at level IV, corresponding to a lymph node as seen on an MRI of the neck in Figure 5 CT, computed tomography; MRI, magnetic resonance imaging

An abscess was also found in the right buccal space, along with a right frontal sinus mucocele. Nasoendoscopy revealed mucopus in the right middle meatus. She underwent a right endoscopic sinus surgery and a biopsy of the right maxillary sinus lesion. Intraoperatively, the right maxillary sinus was filled with pink frond-like tissue, and white-grey concretions with mucopus were noted, as seen in Figure 3. Tissue samples and swab cultures were sent for analysis, and ciprofloxacin was started empirically. Histology revealed squamous dysplasia with areas of invasive SCC. In view of the histology, she underwent magnetic resonance imaging (MRI), which once again revealed a lobulated 4.4 × 3.5 × 3.1 cm hypointense mass in the right maxillary sinus, causing the destruction of the anterior and lateral maxillary walls, with extension into the pre-antral fat and buccal and masticator spaces as depicted in Figure 4. Laterally, there is an involvement of the right temporalis muscle and erosions of the inner aspect of the right zygomatic arch. Superiorly, an erosion of the right orbital floor is noted, with the involvement of the infraorbital canal and extension into the right orbit with close proximity to the right inferior rectus muscle, without obvious involvement. Posteriorly, there is an involvement of the right pterygopalatine fossa. Figure 5 and Figure 6 depict enlarged right level III cervical lymph nodes, suspicious for metastatic nodal spread. She was diagnosed with stage IVA cT3N2bM0 right maxillary sinus SCC and underwent a total maxillectomy and neck dissection and reconstruction with a free anterolateral thigh flap and received adjuvant radiotherapy with no recurrence currently.

Microbiology

Aerobic and anaerobic cultures of intraoperative tissue from the patient’s right maxillary sinus were performed in-house. The specimen was plated onto 5% sheep’s blood agar, chocolate agar, and MacConkey agar and incubated at 37°C in a 5% carbon dioxide incubator, as well as Schaedler agar and phenylethyl alcohol agar with 5% sheep’s blood at 37°C in an anaerobic jar. On day 2 of incubation, a light growth of a non-lactose-fermenting Gram-negative bacillus was observed on primary cultures. The organism was identified as *Salmonella* species by matrix-assisted laser desorption/ionization-time of flight mass spectrometry performed using the microflex® LT (Bruker, Billerica, MA) and MALDI Biotyper® Library (in vitro diagnostic medical device, CE-marked). Serotyping with *Salmonella* antisera (Thermo Fischer Scientific, Waltham, MA) identified the organism as *Salmonella enterica* serovar Enteritidis (I 9:g,m:-). No other pathogens were identified in the routine culture. Disk diffusion susceptibility testing was performed in accordance with Clinical and Laboratory Standards Institute methods and interpretive criteria as described in M100: Performance Standards for Antimicrobial Susceptability Testing (31st edition, 2021) [1]. The isolate was susceptible to ampicillin, ceftriaxone, ciprofloxacin, and sulfamethoxazole-trimethoprim, as seen in Table 1.

**Table 1:** Tissue culture showing organism and culture sensitivities

Antibiotic	Susceptibility
Amoxicillin	Sensitive
Ceftriaxone	Sensitive
Ciprofloxacin	Sensitive
Trimethoprim-sulfamethoxazole	Sensitive

Intravenous amoxicillin/clavulanic acid was administered empirically for eight days, followed by six weeks of ciprofloxacin. The rationale for selecting ciprofloxacin was its narrower spectrum and excellent penetration into the sinus region. Prolonged antibiotic treatment was deemed necessary because the patient was being treated for sinusitis with abscess formation while awaiting further resection for definitive source control, and inflammatory markers remained persistently elevated. Metronidazole was added at the end of the six-week course due to worsening cheek swelling, to empirically cover anaerobic bacteria, which are common pathogens originating from the oral cavity.

## Discussion

*Salmonella *sinusitis is extremely rare, with only three other known cases in the literature, as detailed in Table 2, and this case report presents the first reported instance of *Salmonella* Enteritidis sinusitis in the medical literature [2-4]. The previous dental procedure is likely the risk factor for odontogenic sinusitis due to the close anatomical proximity between the oral cavity and sinuses. However, the common pathogen is usually from the oral flora, including alpha-hemolytic *Streptococcus*, microaerophilic streptococci, *Staphylococcus* *aureus*, and *Streptococcus* *pyogenes*, as well as anaerobes such as *Peptostreptococcus* spp., *Fusobacterium* *nucleatum*, and *Propionibacterium* *acne* [5,6].

**Table 2:** Salmonella-associated sinusitis reported in the literature

Author	Age/sex	Clinical presentation	Microbiology	Treatment	Risk factor	Outcome
Räisänen and Asikainen, 1984 [2]	56/male	Unilateral purulent rhinorrhea	*Salmonella* Typhimurium	Clindamycin, one week; rivampicillin, eight days; irrigation, two	Farmer (animal exposure), however all households and animals tested negative	Cured
Horvath et al., 2016 [3]	29/male	Intermittent fever, pain over the right maxillary, and frontal sinus area	*Salmonella enterica* subspecies *diarizonae*	Ciprofloxacin, three weeks. Subtotal medial maxillectomy with the complete removal of maxillary sinus mucosa	Snake handler (same serotype salmonella isolated from the snake)	Cured
Hartmann, 1964 [4]	70/gender not specified	Enteritis and unilateral purulent rhinorrhea	*Salmonella* Typhimurium (and scanty *Staphylococcus aureus*)	Antibiotics (choice and duration not specified). Caldwell-Luc operation	None found	Cured
Current case	77/female	Unilateral tooth and cheek pain with worsening symptoms after tooth extraction	*Salmonella* Enteritidis. Other organisms isolated from superficial cultures: scanty *Klebsiella variicola*, *Stenotrophomonas maltophilia*, and mixed anaerobes	Amoxicillin-clavulanate, followed by ciprofloxacin and metronidazole for ~7 weeks until total maxillectomy	Concurrent maxillary squamous cell carcinoma, possibly kombucha related	Cured

In our case, the consumption of potentially contaminated kombucha might have introduced pathogens into the oral cavity, subsequently leading to direct transmission to the right maxillary sinus. Alternatively, hematogenous transmission may have occurred, involving gut-derived bacteremia and the subsequent tissue invasion of the right maxillary sinus. The underlying sinus squamous cell carcinoma likely contributed to the increased risk of infection by altering the local anatomy and microenvironment, facilitating pathogen colonization and invasion.

Kombucha is produced by fermenting sweetened tea using a starter culture of acetic acid bacteria, lactic acid bacteria, and yeast [7]. While there is no definitive evidence linking kombucha to *Salmonella* infections or outbreaks, the patient’s kombucha was home-brewed, which may present a contamination risk if inadequate hygiene precautions were undertaken. Even though the low pH of fermented kombucha is expected to inhibit the growth of food-borne pathogens, a study investigating the survival of *Salmonella* and Shiga toxin-producing *Escherichia coli *in kombucha found that low levels of residual *Salmonella* could still be detected in some preparations even after 14 days of fermentation [7]. In this case, the laboratory culture of the patient’s kombucha starter samples identified no microbial growth, although specimen deterioration or nonrepresentative sampling cannot be ruled out.

## Conclusions

We report a rare and noteworthy case of *Salmonella *Enteritidis sinusitis in conjunction with a right maxillary sinus squamous cell carcinoma. Our findings underscore the importance of considering atypical presentations in clinical practice. *Salmonella* is a very rare and poorly understood cause of sinusitis, with scarce cases in literature. Traditional risk factors include transmission through contaminated food, water, or animals. Further research is needed to explore the causal relationship between kombucha consumption and *Salmonella *infections.
